# The synthetic Tie2 agonist peptide vasculotide protects against vascular leakage and reduces mortality in murine abdominal sepsis

**DOI:** 10.1186/cc10523

**Published:** 2011-10-31

**Authors:** Philipp Kümpers, Faikah Gueler, Sascha David, Paul Van Slyke, Daniel J Dumont, Joon-Keun Park, Clemens L Bockmeyer, Samir M Parikh, Hermann Pavenstädt, Hermann Haller, Nelli Shushakova

**Affiliations:** 1 Department of Medicine D, Division of General Internal Medicine, Nephrology, and Rheumatology, University Hospital Münster, Albert-Schweitzer-Strasse 33, Münster 48149 Germany; 2 Department of Nephrology and Hypertension, Hannover Medical School, Carl-Neuberg Strasse 1, Hannover 30625, Germany; 3 Center of Vascular Biology Research, Beth Israel Deaconess Medical Center, Harvard Medical School, 99 Brookline Avenue, Boston, MA 02215, USA; 4 Molecular and Cellular Biology Research, Sunnybrook Research Institute, and Department of Medical Biophysics, University of Toronto, 2075 Bayview Avenue, Toronto, ON M4N 3M5, Canada; 5 Institute of Pathology, Hannover Medical School, Carl-Neuberg-Strasse 1, Hannover 30625, Germany

## Abstract

**Introduction:**

Angiopoietin-1 (Angpt1), the natural agonist ligand for the endothelial Tie2 receptor, is a non-redundant endothelial survival and vascular stabilization factor that reduces endothelial permeability and inhibits leukocyte-endothelium interactions. Here we evaluate the efficacy of a novel polyethylene glycol (PEG)-clustered Tie2 agonist peptide, vasculotide (VT), to protect against vascular leakage and mortality in a murine model of polymicrobial abdominal sepsis.

**Methods:**

Polymicrobial abdominal sepsis in C57BL6 mice was induced by cecal-ligation-and-puncture (CLP). Mice were treated with different dosages of VT or equal volume of phosphate-buffered saline (PBS). Sham-operated animals served as time-matched controls.

**Results:**

Systemic administration of VT induced long-lasting Tie2 activation *in vivo*. VT protected against sepsis-induced endothelial barrier dysfunction, as evidenced by attenuation of vascular leakage and leukocyte transmigration into the peritoneal cavity. Histological analysis revealed that VT treatment ameliorated leukocyte infiltration in kidneys of septic mice, probably due to reduced endothelial adhesion molecule expression. VT-driven effects were associated with significantly improved organ function and reduced circulating cytokine levels. The endothelial-specific action of VT was supported by additional *in vitro *studies showing no effect of VT on either cytokine release from isolated peritoneal macrophages, or migratory capacity of isolated neutrophils. Finally, administration of VT pre-CLP (hazard ratio 0.39 [95% confidence interval 0.19-0.81] *P *< 0.001) and post-CLP reduced mortality in septic mice (HR 0.22 [95% CI 0.06-0.83] *P *< 0.05).

**Conclusions:**

We provide proof of principle in support of the efficacious use of PEGylated VT, a drug-like Tie2 receptor agonist, to counteract microvascular endothelial barrier dysfunction and reduce mortality in a clinically relevant murine sepsis model. Further studies are needed to pave the road for clinical application of this therapeutic concept.

## Introduction

In 1995/1996, Sato and colleagues [1] and Davis and colleagues [2] discovered Tie2 and its agonist ligand angiopoietin-1 (Angpt1) as the second class of vascular-specific receptor tyrosine kinases; the first was the vascular endothelial growth factor (VEGF)/VEGF receptor system. Studies in Angpt1^-/- ^and Tie2^-/- ^knockout mice, which die *in utero *owing to severe vascular remodeling, convincingly demonstrated the importance of operational Angpt1/Tie2 signaling for developmental angiogenesis [1,3]. Besides having a role for vascular integrity in growing mice, Angpt1 was subsequently identified as a potent anti-permeability factor that protected the vasculature of adult mice from plasma leakage induced by VEGF and other inflammatory stimuli [4]. Given the absence of redundant systems to bypass the function of Angpt1/Tie2, it was speculated early that excess Angpt1 effectively abolishes microvascular leakage in experimental sepsis. Indeed, subsequent studies confirmed the latter hypothesis by demonstrating that either acute administration of recombinant Angpt1 protein or gene transfer of Angpt1 prevented capillary leakage, protected against subsequent acute kidney injury (AKI) and acute lung injury (ALI), and improved survival in Gram-negative murine endotoxemia [5-11].

During human endotoxemia and sepsis, circulating Angpt1 levels remain unchanged or even decrease, whereas the endogenous context-specific Tie-2 antagonist, angiopoietin-2 (Angpt2), is rapidly released by the activated endothelium and disrupts the constitutive Angpt1/Tie2 signaling by preventing Angpt1 from binding to the receptor [12-19].

We and others have shown that Angpt2 levels in plasma from critically ill patients with sepsis correlate with the extent of pulmonary vascular leakage in ALI [19], increase with the severity of AKI [16], and independently predict mortality in the intensive care unit [14,16,20-22]. Of note, local or systemic injection of recombinant Angpt2 in otherwise-healthy mice is sufficient to provoke tissue edema or pulmonary vascular leakage, respectively [23,24].

Consistent with these observations, agents that activate the endothelial-specific Tie2 receptor pathway and sufficiently protect against capillary leakage, vascular inflammation, and subsequent multiple-organ damage are highly desirable for the treatment of patients with sepsis. However, neither gene therapy (with Angpt1) nor the administration of large doses of recombinant Angpt1 protein is feasible in clinical routine [6].

Recently, Tournaire and colleagues [25] described the discovery of a short synthetic peptide (HHHRHSF) that binds with high affinity to the extracellular portion of the Tie2 receptor but lacks the capacity to displace either Angpt1 or Angpt2. Using this peptide clustered as a tetramer by way of avidin/biotin, Van Slyke and colleagues [26] demonstrated that Tie2 could be activated in a manner analogous to Angpt1. Subsequently, this proof-of-principle compound, termed vasculotide (VT), was reengineered into a more pharmaceutically amenable preparation that excludes the avidin/biotin complex in favor of a tetrameric polyethylene glycol (PEG) scaffold (Additional file 1).

We hypothesized that systemic administration of PEGylated VT would activate Tie2 in animals, protect against vascular leakage and tissue injury, and improve survival in a murine cecal ligation and puncture (CLP) model of polymicrobial sepsis. We performed studies in mice, resident peritoneal macrophages (PMs), and isolated bone marrow-derived neutrophils to test this hypothesis.

## Materials and methods

### Synthesis, purification, and validation of vasculotide

Peptide NH_2_-CHHHRHSF-COOH (GenScript USA Inc., Piscataway, NJ, USA) was reacted with four-armed PEG (polydisperse, average molecular weight of 10 kDa) (Sunbright PTE-100MA; NOF Corporation, Tokyo, Japan) in a 12:1 molar ratio. Covalent attachment of the peptide to the activated maleimide groups took place in phosphate-buffered saline (PBS), pH 6.4, at room temperature with agitation for 4 hours. Products were purified by way of dialysis (Slide-A-Lyzer, 7000 Da, MWCO; Pierce Biotechnology, now part of Thermo Fisher Scientific Inc., Rockford, IL, USA) at 4°C with stirring. Products were first dialyzed against PBS at pH 7.4 (two exchanges, 300X volume, 3 hours each) and then double-distilled water (eight exchanges, 300X volume, 6 hours each). Dialyzed products were then frozen, lyophilized, and weighed to calculate product yield. Validation of VT was assessed by MALDI TOF (matrix-assisted laser desorption/ionization time-of-flight mass spectrometer) to determine the potential presence of free peptide impurities and to measure the molecular size distribution of the final product (efficiency of conjugation).

### *In vivo *animal studies

All procedures were approved by the local committee for care and use of laboratory animals (Lower Saxony Office for Consumer Protection and Food Safety) and were performed in accordance with international guidelines on animal experimentation. Eight- to 10-week-old male C57BL6 mice (20 to 25 g) were obtained from Charles River Laboratories (Sulzfeld, Germany). Mice were maintained on mouse chow and tap water *ad libitum *in a temperature-controlled chamber at 24°C with a 12:12-hour light-dark cycle. Polymicrobial sepsis in mice was induced by CLP. In brief, mice were anesthetized with isofluorane (induction of 3%, maintenance of 1.5%, and oxygen flow of 3 L/minute), and a 1-cm ventral midline abdominal incision was made. The cecum was then exposed, ligated with 4-0 silk sutures just distal to the ileocecal valve (comprising 70% of the cecum and sparing the cecal vessels) to avoid intestinal obstruction, and punctured through with a 24-gauge needle. The punctured cecum was gently squeezed to expel a 1- to 2-mm droplet of fecal material and returned to the abdominal cavity. The incision was then closed in layers with 4-0 surgical sutures. Mice were fluid-resuscitated with prewarmed normal saline (500 μL) intraperitoneally (i.p.) immediately after the procedure. Sham animals underwent the same procedure except for CLP. All experiments were carried out at the same time of day.

### Survival analysis

Mice were pretreated i.p. with different dosages (50, 200, or 500 ng) of VT or PBS at 16 hours and 1 hour prior to CLP or sham surgery and at 24 hours and 48 hours afterward. To test whether delayed administration of VT improves survival in established sepsis, a second group of mice was subjected to CLP first and then treated with 200 ng of VT or PBS i.p. at 2, 24, and 48 hours after CLP surgery. Survival time was recorded daily up to 14 days after CLP or sham surgery.

### Short-term experiments: peritoneal lavage and Evans blue permeability assay

In short-term experiments, mice were pretreated i.p. with 200 ng of VT or PBS at 16 hours and 1 hour prior to surgery. Immediately after surgery, 0.25% wt/vol Evans blue dye (200 μL) was injected intravenously. Evans blue dye avidly binds to serum albumin and therefore can be used as a tracer for macromolecule flux across the microvasculature. At 18 hours after CLP or sham operation, mice were anesthetized with isofluorane for blood sampling. Subsequently, animals were sacrificed and peritoneal lavage (PL) was performed with 3 mL of PBS. The volume of the collected PL was measured in each sample, and the total cell count was assessed with a hemocytometer (Neubauer Zählkammer, Gehrden, Germany). For quantification of polymorphonuclear neutrophil (PMN) accumulation, differential cell counts were performed on cytospins (10 minutes at 55*g*) stained with hematoxylin and eosin. PL fluids were centrifuged at 500*g *for 5 minutes to pellet cells, and cell-free supernatants were frozen at -70°C for subsequent measurement of proinflammatory mediators. The concentration of Evans blue dye in appropriate dilutions of serum and PL fluids was measured spectrophotometrically at 620 nm. The following formula was used to correct the optical densities for contamination with heme pigments: E_620 (corrected) _= E_620 (raw) _- (E_405 (raw) _× 0.014). Plasma exudation was quantitated as the ratio of extinction in PL fluid to extinction in plasma.

### Blood and tissue sampling

Mice were anesthetized with isofluorane for blood sampling and subsequently sacrificed for tissue sampling at 18 hours after CLP or sham surgery (n = 10 per group). Blood samples were obtained from the cavernous sinus by means of a capillary. Kidneys were removed and either fixed for 20 hours in 3.75% paraformaldehyde in Sörensen's phosphate buffer and embedded in paraffin for histologic examination or snap-frozen in isopentane (-40°C) for cryostat sectioning.

### Clinical chemistry

Serum level of creatinine and urea and the activities of aspartate aminotransferase (AST) and alanine aminotransferase (ALT) were measured by an automated method and an Olympus AU 400 analyzer (Beckman Coulter Inc., Krefeld, Germany).

### Cytokine detection in serum and peritoneal lavage samples

Serum levels of the proinflammatory cytokines tumor necrosis factor-alpha (TNF-α), interleukin-6 (IL-6), and macrophage chemoattractant protein-1 (MCP-1) were quantified by bead-based flow cytometry assay (CBA Kit; BD Biosciences, Heidelberg, Germany) in accordance with the instructions of the manufacturer. The concentrations of two major neutrophil chemoattractants [27], macrophage inflammatory protein-2 (MIP-2) and keratinocyte chemoattractant (KC), in serum and PL fluids were measured with the respective specific enzyme-linked immunosorbent assay (ELISA) kits (R&D Systems, Wiesbaden, Germany) in accordance with the instructions of the manufacturer.

### Immunofluorescence

Immunofluorescence was performed on ice-cold acetone-fixed cryosections (6 μm) by using the following primary antibodies: rat anti-mouse intercellular adhesion molecule-1 (ICAM-1) (or CD54, clone KAT-1; AbD Serotec, Oxford, UK), rat anti-mouse vascular cell adhesion molecule-1 (VCAM-1) (or CD106, clone MVCAM.A; AbD Serotec), rat anti-mouse Gr-1-positive neutrophils (clone 7/4; AbD Serotec), and rabbit anti-human/mouse phosphorylated Tie2 (pTie2, Y1102; R&D Systems, Oxon, UK). Non-specific binding sites were blocked with 10% normal donkey serum (Jackson ImmunoResearch Laboratories, Inc., West Grove, PA, USA) for 30 minutes. Thereafter, sections were incubated with the primary antibody for 1 hour. All incubations were performed in a humid chamber at room temperature. For fluorescent visualization of bound primary antibodies, sections were further incubated with Cy3-conjugated secondary antibodies (Jackson ImmunoResearch Laboratories, Inc.) for 1 hour in the dark. For negative controls, the staining procedure was performed as described without the primary antibodies. Specimens were analyzed with a Zeiss Axioplan-2 imaging microscope and the digital image-processing program AxioVision 4.6 (Zeiss, Jena, Germany).

Analysis of infiltrating neutrophils was done by enumerating Gr-1-positve cells in kidney tissue sections (n = 7 to 10 per group). Data are expressed as the mean number of 20 randomly chosen, non-overlapping fields per section. Analysis of pTie2 expression in renal vasculature was done by semiquantitative scoring as follows: 0 = no, 1 = very weak, 2 = weak, 3 = moderate, 4 = strong, and 5 = very strong expression. Data are expressed as the mean the mean score of 20 randomly chosen, non-overlapping fields per section. Analysis of adhesion molecule expression in specific vascular sections (that is, glomeruli, arteries, and peritubular capillaries) was done by semiquantitative scoring as follows: 0 = no, 1 = very weak, 2 = weak, 3 = moderate, 4 = strong, and 5 = very strong expression. Data are expressed as the mean score of 40 glomeruli or 20 randomly chosen, non-overlapping interstitial fields (for arteries and peritubular capillaries) per section. The investigator had no knowledge of the treatment group assignment.

### Tie2 immunoprecipitation

For determination of Tie2 phosphorylation, kidneys from healthy mice (n = 3 per group) were harvested at 16 and 34 hours after VT pretreatment (200 ng of VT i.p. at 0 and 16 hours). Mice (n = 3) injected with control buffer (PBS) only served as baseline controls (0 hours). Kidney tissue from each animal was homogenized in RIPA lysis buffer (50 mM Tris-HCl pH 7.5, 150 mM NaCl, 1% Igepal, 0.5% sodium deoxycholate, 0.1% SDS, 2 mM sodium orthovanadate, and protease inhibitors). Lysates were then centrifuged at 14,000*g *for 15 minutes at 4°C. The supernatants were aliquoted on the basis of the protein concentration measured by using a BCA (bicinchoninic acid) protein assay (Thermo Fisher Scientific Inc.) and stored at -80°C. Tie2 was immunoprecipitated from the supernatant by using 2 to 4 μg of anti-Tie2 antibody C20 (Santa Cruz Biotechnology, Inc., Santa Cruz, CA, USA) that had been precoupled to 25 μL of protein-A-Sepharose (GE Healthcare, Little Chalfont, Buckinghamshire, UK) [28].

### Tie2 immunoblotting

Proteins were resolved on a 7.5% polyacrylamide gel and transferred to PVDF (polyvinylidene fluoride) (PerkinElmer Life and Analytical Sciences, Inc., Waltham, MA, USA) membranes. The membranes were blocked for 1 hour at room temperature in 5% bovine serum albumin (BSA) prior to detecting phosphotyrosine or in 5% non-fat dry milk for detecting total Tie2. The membranes were incubated with 1 μg/mL anti-phosphotyrosine antibody 4G10 (Upstate/Millipore, Billerica, MA, USA) or 0.5 μg/mL anti-Tie2 monoclonal antibody (Millipore). Proteins were visualized by using secondary antibodies conjugated to HRP (horseradish peroxidase) (Bio-Rad, Mississauga, ON, Canada) followed by enhanced chemiluminescence [28].

### Functional analysis of resident peritoneal macrophages *in vitro*

Mouse resident PMs were obtained from untreated wild-type mice by PL by using 10 mL of PBS (2 × 5 mL). The cells were centrifuged at 300*g *for 10 minutes at 4°C, the supernatants were decanted, and the cell pellets were washed twice with RPMI 1640 medium. Cells were resuspended in RPMI 1640 medium supplemented with 10% fetal calf serum (FCS) and plated in 24-well plastic culture plates (Corning Incorporated, Corning, NY, USA) to achieve a final concentration of 1.5 × 10^6 ^cells/mL per well. The plates were incubated for 2 hours at 37°C, 5% CO_2_, and 95% humidity to allow macrophage adherence. Non-adherent cells were removed by vigorous washing with RPMI 1640 medium. Primary murine PMs were starved for 24 hours in 1% FCS/RPMI 1640 medium in 24-well plates and preincubated or not with 25 or 100 ng/mL VT for 1 hour and then stimulated with 10 ng/mL lipopolysaccharides (LPSs) (O111:B4, Sigma-Aldrich, Munich, Germany) for 4 hours. In some experiments, PMs were preincubated with a molar excess (5 μg/mL) of a recombinant human Tie2/Fc chimera (R&D Systems, Wiesbaden, Germany) for 30 minutes prior to the addition of VT. The medium was collected from cultured PMs with or without the treatments mentioned above and centrifuged. Production of MIP-2 and KC was examined in supernatants by using specific ELISA kits (R&D Systems) in accordance with the instructions of the manufacturer.

### Chemotaxis assay *in vitro*

Wild-type murine bone marrow cells were suspended at 7.5 × 10^6 ^cells per mL RPMI 1640 medium with 0.25% BSA (Sigma-Aldrich). Neutrophils in the cell suspension were determined by means of a Vet ABC hematology analyzer (scil animal care company, Gurnee, IL, USA) to be in the range of 64% to 68% of total cells. Next, 100 μL of the cell suspension was placed into the upper well insert of a 6.5-mm diameter, 3-μm pore polycarbonate Transwell chemotaxis chamber (Costar Corning, Corning, NY, USA), and the bottom well was filled with 600 μL of RPMI 1640/0.25% BSA (medium control). PMNs were preincubated with a different concentration of VT for 1 hour and thereafter used for Transwell migration assays toward an optimal dose (50 ng/mL) of recombinant human C5a (rhC5a; Sigma-Aldrich) or toward PL pools (diluted 1:2 with RPMI 1640/0.5% BSA) obtained at 18 hours after CLP surgery. The chambers were incubated at 37°C and 5% CO_2 _for 2 hours. Next, 50 μL of 70 mM EDTA (ethylenediaminetetraacetic acid) solution was added to the bottom chambers to release adherent cells from the lower surface of the membrane and from the bottom of the well. Plates were further incubated for 30 minutes at 4°C, inserts were removed, and the transmigrated neutrophils were vigorously suspended and counted by using a Vet ABC hematology analyzer. Migration of PMNs from the insert to the bottom well was quantified as the percentage of total PMNs loaded into the upper chamber.

### Statistical analysis

Data are presented as mean ± standard error of mean. Multiple comparisons were analyzed for significant differences by using the one-way analysis of variance with the Tukey as a *post hoc *test for multiple comparisons. Kaplan-Meier plots were used to illustrate survival between treatment groups, and statistical assessment was performed by the log-rank test. Animals still alive at 14 days after CLP were censored at day 14. All tests were two-sided, and significance was accepted at a *P *value of less than 0.05. GraphPad Prism version 5.02 (GraphPad Prism Software Inc., San Diego, CA, USA) was used for data analysis and figure preparation.

## Results

### Vasculotide induces Tie2 posphorylation *in vivo*

Having verified that VT induces Tie2 phosphorylation (that is, activation) *in vitro *in previous work [26], we initially tested whether VT can also induce Tie2 activation *in vivo*. We performed immunoprecipitation for Tie2 and consecutive immunoblot for phospho-tyrosine (pY) in kidney homogenates obtained from healthy C57BL6 mice treated with 200 ng of VT i.p. As expected, VT treatment induced a long-lasting increase of the phosporylated fraction of Tie2 *in vivo *(Additional file 1).

### Vasculotide prevents capillary leakage and neutrophil transmigration *in vivo*

**Figure 1:**
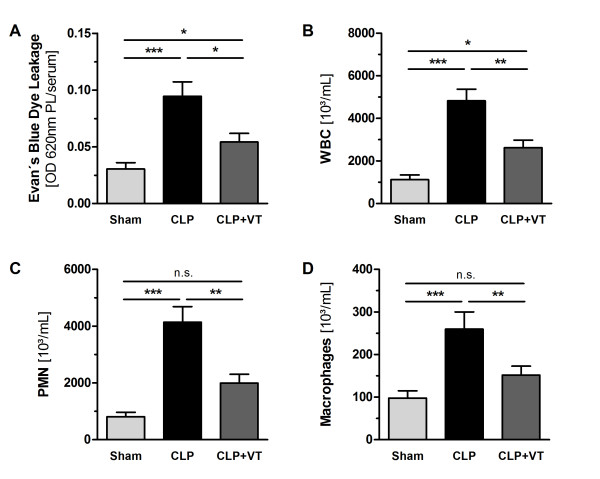
**Vasculotide (VT) prevents capillary leakage and neutrophil transmigration *in vivo***. Mice were pretreated with VT (200 ng intraperitoneally) or phosphate-buffered saline (PBS) at 16 hours and 1 hour prior to cecal ligation and puncture (CLP). Sham animals were pretreated with PBS at 16 hours and 1 hour prior to surgery. Immediately after surgery, Evans blue dye was injected intravenously. **(A) **Evans blue dye content in peritoneal lavage (PL) fluid at 18 hours after CLP or sham operation. **(B) **White blood cell (WBC) counts and **(C, D) **differential cell counts from PL samples at 18 hours after CLP or sham operation. Data are expressed as mean ± standard error of the mean (n = 7 to 10 mice per group). *P < 0.05; **P < 0.01; **P < 0.001. n.s., not significant; OD, optical density; PMN, polymorphonuclear neutrophil.

To assess the putative anti-permeability and anti-transmigratory capacity of VT *in vivo*, we quantified the extravasation of Evans blue dye and the transmigration of leukocytes into the abdominal cavity. After initial dose-ranging studies, C57BL6 mice received 200 ng of VT (or an equal volume of PBS) i.p. at 16 hours and 1 hour prior to CLP or sham surgery, respectively. Compared with sham surgery, CLP produced a significant increase in dye extravasation at 18 hours after the procedure. Of note, sepsis-induced peritoneal hyperpermeability was substantially reduced by pretreatment with VT (Figure 1A). Consistent with the scarcity of early ALI in the CLP model used here, Evans blue dye content was not different in bronchoalveolar lavage fluid samples from CLP, CLP plus VT, or sham animals (data not shown). At 18 hours after CLP, the total inflammatory cell count in the PL fluid was increased approximately fivefold over sham controls, and this was attributable mainly to an increase in PMNs (~86%) (Figure 1B, C). VT pretreatment significantly blocked sepsis-induced PMN transmigration, as evidenced by reduced total cell counts and PMNs in the PL fluid. In addition, macrophage counts in the PL fluid were significantly reduced by VT treatment (Figure 1D).

### Vasculotide ameliorates endothelial adhesion molecule expression and leukocyte influx in septic kidneys

**Figure 2:**
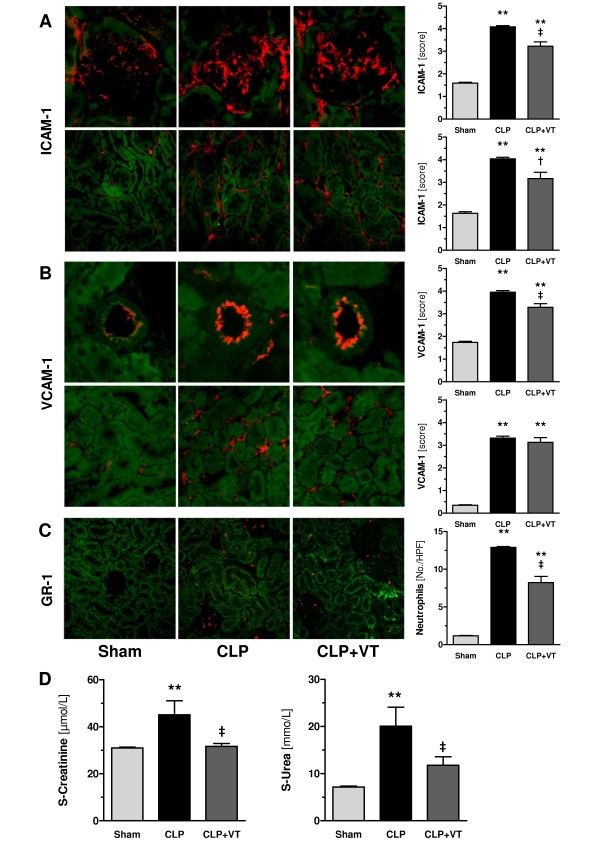
**Vasculotide (VT) ameliorates acute kidney injury**. Mice were pretreated with VT (200 ng intraperitoneally) or phosphate-buffered saline at 16 hours and 1 hour prior to cecal ligation and puncture (CLP) or sham surgery, respectively. Kidney tissues were harvested at 18 hours after CLP or sham treatment. **(A) **Immunofluorescence study of intercellular adhesion molecule-1 (ICAM-1) and **(B) **vascular cell adhesion molecule-1 (VCAM-1) protein expression (red) among specific sections within the renal vasculature (that is, glomeruli [400 ×] arteries and peritubular capillaries [200 ×]). **(C) **Immunofluorescence study of infiltrating neutrophils (red) in Gr-1-stained sections (200 ×). Bar charts show the results of semiquantitative scoring results (see Materials and methods). **(D) **Bar charts of serum levels of serum creatinine and urea. Data are expressed as mean ± standard error of the mean (n = 7 to 10 mice per group). **P < 0.01 versus sham. †*P *< 0.05; ‡*P *< 0.01 versus CLP without VT. HPF, high-power field.

Leukocyte trafficking across the vascular endothelium is critically dependent on endothelial adhesion molecules, such as ICAM-1, VCAM-1, and E-selectin [29,30]. Therefore, we investigated whether VT ameliorates upregulation of endothelial adhesion molecules and subsequent leukocyte influx in vital organs, such as the kidney. We used immunofluorescence staining to localize and semiquantify the protein expression of ICAM-1 and VCAM-1 among specific vascular sections within the renal vasculature. CLP-induced upregulation of ICAM-1 in glomeruli and peritubular capillaries was significantly reduced in VT-treated mice compared with buffer-treated controls at 18 hours after CLP (Figure 2A). CLP-induced upregulation of VCAM-1 expression did not occur in the glomerular endothelium (data not shown) but was present in arteries and, to a lesser extent, in peritubular capillaries. In contrast to septic controls, VT significantly reduced arterial VCAM-1 expression but did not ameliorate upregulation of VCAM-1 in peritubular capillaries after CLP (Figure 2B). Despite repeated attempts, we were unable to stain for E-selectin and to quantify ICAM-1 or VCAM-1 expression by Western blotting. As shown by semiquantitative analysis, VT treatment resulted in considerable attenuation of leukocyte influx into septic kidneys at 18 hours after CLP (Figure 2C). Consistent with immunofluorescence data, VT-treated mice were largely protected from acute liver injury (Additional file 2) and AKI compared with buffer-treated CLP mice at 18 hours after CLP induction, as evidenced by significantly lower serum creatinine and urea levels (Figure 2D).

### Vasculotide augments Tie2 signaling *in vivo*

**Figure 3:**
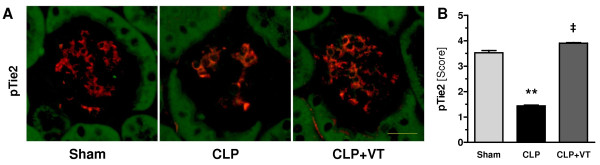
**Vasculotide (VT) augments Tie2 signaling *in vivo***. Mice were pretreated with VT 200 ng intraperitoneally) or phosphate-buffered saline at 16 hours and 1 hour prior to cecal ligation and puncture (CLP) or sham surgery, respectively. Kidney tissues were harvested at 18 hours after CLP or sham treatment. **(A) **Immunofluorescence study of phosphorylated Tie2 (pTie2, red) within glomeruli (400 ×). **(B) **Bar charts showing the results of semiquantitative scoring within the renal vasculature (see Materials and methods). Data are expressed as mean ± standard error of the mean (n *= *7 to 10 mice per group). **P < 0.01 versus sham. ‡*P *< 0.01 versus CLP without VT.

We recently reported a reduction of Tie2 transcript and protein abundance in septic kidneys, which is proportional to the severity of the CLP model used [6]. To explore signaling changes induced by CLP and VT, we used immunofluorescence to detect and quantify pTie2 (that is, activated Tie2) in kidneys from septic and sham-treated mice. CLP resulted in considerable attenuation of pTie2 abundance (that is, deactivation) compared with sham operation. VT pretreatment fully restored pTie2 staining to control intensity (Figure 3). Together, these results suggest that the decreased Tie2 protein abundance induced by CLP is overcome by increased activation of the remaining Tie2 molecules when VT is given.

### Vasculotide suppresses CLP-induced upregulation of proinflammatory and chemotactic cytokines

**Figure 4:**
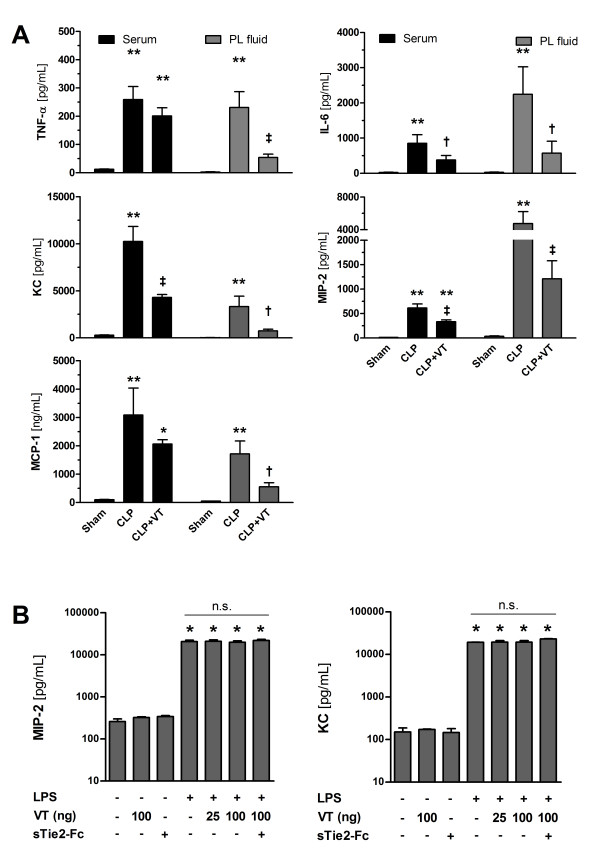
**Vasculotide (VT) suppresses cecal ligation and puncture (CLP)-induced upregulation of proinflammatory cytokines *in vivo *but does not affect cytokine release from macrophages *in vitro***. **(A) **Mice were pretreated with VT (200 ng intraperitoneally) or phosphate-buffered saline (PBS) at 16 hours and 1 hour prior to CLP or sham surgery, respectively. Levels of tumor necrosis factor-alpha (TNF-α), interleukin-6 (IL-6), keratinocyte-derived chemokine/interleukin-8 homologue (KC), macrophage inflammatory protein-2 (MIP-2), and monocyte chemotactic protein-1 (MCP-1) in serum and peritoneal lavage (PL) fluid samples at 18 hours after CLP or sham surgery were quantified by bead-based flow cytometry or enzyme-linked immunosorbent assay (ELISA), respectively. Data are expressed as mean ± standard error of the mean (SEM) (n = 7 to 10 mice per group). *P < 0.05; **P < 0.01 versus sham. †*P *< 0.05; ‡*P *< 0.01 versus CLP without VT. **(B) **Mouse resident peritoneal macrophages were obtained from untreated wild-type mice by PL. Cells were cultured in RPMI 1640/1% fetal calf serum (FCS), preincubated or not with 25 or 100 ng/mL VT for 1 hour, and then stimulated with lipopolysaccharide (LPS) (10 ng/mL) for 4 hours. In some experiments, peritoneal macrophages were preincubated with an sTie-2-Fc chimera (5 μg/mL) 30 minutes prior to the addition of VT. (A) MIP-2 and (B) KC protein levels were analyzed in conditioned medium by specific ELISA. Results are expressed as the mean ± SEM from three independent experiments performed in triplicate. *P < 0.001 versus unstimulated control.

The magnitude of the inflammatory response is also dependent on local and systemic production of proinflammatory as well as chemotactic mediators: early-response cytokines, such as TNF-α and IL-6, induce endothelial activation and enhance endothelial adhesion molecule expression, whereas chemokines, especially KC, MIP-2, and MCP-1, play a central role in inflammatory cell chemotaxis by attracting and stimulating specific subsets of leukocytes. As expected, both cytokines and chemokines were significantly elevated in serum and PL fluid at 18 hours after CLP (Figure 4A). Pretreatment with 200 ng of VT caused a significant reduction in serum and PL fluid levels in each of the aforementioned cytokines-except for serum TNF-α, which, however, was not affected by VT-at 18 hours (Figure 4A).

### Vasculotide affects neither cytokine release from resident peritoneal macrophages nor migratory capacity of polymorphonuclear neutrophils *in vitro*

Gu and colleagues [31] recently reported that Tie2 activation by Angpt1 is sufficient to block LPS-induced cytokine production in a macrophage cell line. Tie2 expression in resident PMs was confirmed at the protein level by ELISA and Western blotting (data not shown). Next, we tested whether VT interferes with the LPS-induced release of MIP-2 and KC from resident PMs *in vitro*. Stimulation of PMs isolated from healthy C57BL6 mice with LPS resulted in a strong release of levels of both KC and MIP-2. However, preincubation of the cells with a different concentration of VT was not able to change this response (Figure 4B), indicating that the attenuation of PMN recruitment after VT treatment *in vivo *is not attributable to changes in the *net *release of cytokines from activated macrophages.

In addition, we tested whether VT attenuates PMN migration *in vitro *in a direct fashion. Migratory capacity of isolated PMNs from healthy C57BL6 mice elicited with PL fluid or rhC5a was assessed by Transwell migration assay. Pooled PL fluid from PBS-treated septic mice obtained at 18 hours after CLP elicited a 10-fold increase in PMN migration in comparison with PL fluid from sham-treated controls. This response was significantly less pronounced (~40%) if PL fluid from VT-treated septic mice was used and reached only sixfold over controls in PMN migration (Additional file 3). Interestingly, preincubation of PMNs with a different concentration of VT for 1 hour did not reduce PMN migration toward rhC5a *in vitro*. Similarly, preincubation of PMNs with VT *in vitro *was not sufficient to reduce PMN migration toward PL fluid from PBS-treated septic mice (Additional file 3). Collectively, these data suggest that VT does not affect PMN migration in a direct fashion at the cellular level but that VT is sufficient to attenuate PMN accumulation *in vivo*.

### Vasculotide improves survival of septic mice

**Figure 5:**
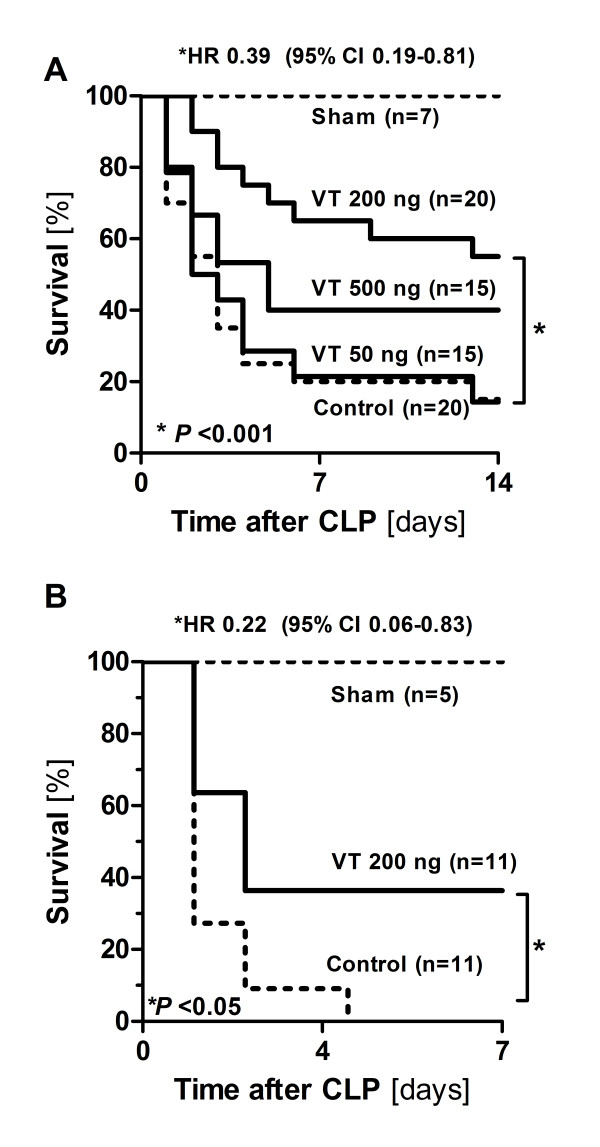
**Vasculotide (VT) improves survival of septic mice**. Kaplan-Meier curves with log-rank testing show the effect of VT on survival in polymicrobial sepsis induced by cecal ligation and puncture (CLP). **(A) **Mice were pretreated with different dosages (50, 200, or 500 ng) of VT or phosphate-buffered saline (PBS) intraperitoneally (control) at 16 hours and 1 hour prior to CLP and at 24 hours and 48 hours afterward. **(B) **Mice were subjected to CLP first and then treated with 200 ng of VT or PBS intraperitoneally at 2, 24, and 48 hours after CLP. Sham animals underwent the same procedure as CLP mice except for CLP and were treated with control buffer (PBS). CI, confidence interval; HR, hazard ratio.

Lastly, we performed survival experiments, the benchmark of any successful treatment regimen in human sepsis. C57BL6 mice received 50, 200, or 500 ng of VT (or an equal volume of PBS) i.p. at 16 hours and 1 hour prior to CLP or sham surgery and at 24 hours and 48 hours after surgery. As a result, only 15% of mice pretreated with either PBS or 50 ng of VT survived compared with 55% and 40% in the 200 ng and 500 ng VT groups, respectively. Pretreatment with 200 ng of VT, in comparison with PBS, was significantly associated with better outcome (hazard ratio 0.39, 95% confidence interval 0.19 to 0.81; *P *< 0.001) (Figure 5A). Finally, we tested whether delayed administration of VT improves survival in early sepsis. Therefore, mice were subjected to CLP first and then treated with 200 ng of VT i.p. at 2, 24, and 48 hours after surgery. As a result, 100% of mice in the control group died whereas 36% of mice survived after rescue treatment with 200 ng of VT (hazard ratio 0.22, 95% confidence interval 0.06 to 0.83; *P *< 0.05) (Figure 5B).

## Discussion

In sepsis, there is a fine line between overwhelming the offending pathogen and inflicting severe collateral damage to the host. In particular, disseminated (that is, systemic) endothelial activation/injury is a devastating but common feature of the hosts' (often disproportionate) immune response. It is characterized by capillary leakage with subsequent tissue edema, distributive shock, and leukocyte infiltration of vital remote organs [29,30]. Despite advances in understanding the crucial role of the vasculature in sepsis pathophysiology, the mainstay of treatment remains non-specific supportive care [32]. Pharmacologic strategies aimed at specifically strengthening the vascular barrier thus could offer resistance to leakage-associated conditions, such as ALI, AKI, and multiple organ dysfunction syndrome.

Since the original report by Witzenbichler and colleagues [11] in 2005, several studies have confirmed that activation of the non-redundant and endothelial-specific Tie2 receptor pathway by adenoviral overexpression of its agonist ligand Angpt1 (AdAngpt1) is sufficient to prevent endotoxemia-induced vascular leakage [8] and subsequent AKI [9] and ALI [5] in rodents. In a clinical scenario, however, systemic delivery of Angpt1 by using viral vectors is not yet feasible, and this is due in part to the acuity of the disease state and the fact that it would take at least 2 to 3 days to reach the peak plasma concentration of Angpt1. Consistent with this notion, Huang and colleagues [5] found that administration of AdAngpt1 shortly after endotoxin exposure failed to improve outcome. Moreover, all of the aforementioned studies used LPS from a well-defined bacterial strain to bring about the onset of sepsis, whereas in human sepsis, the pathogenic bacteria are often not known, and mixed infections that involve both Gram-negative and Gram-positive bacteria are common [33].

To circumvent these issues, we recently investigated whether acute intravenous administration of the recombinant form of human Angpt1 protein could protect against multiple organ dysfunction syndrome in a murine model of polymicrobial abdominal sepsis [6]. Although we could indeed reproduce most of the findings pursued with virally delivered Angpt1, histologic studies by Cho and colleagues [34] suggest that intravenously administered native Angpt1, in addition to its rather short protein half-life, might get stuck to macrovascular endothelial cells and probably does not reach the microvasculature efficiently. Moreover, large-scale production of recombinant Angpt1 is costly and is hindered by the aggregation and insolubility of the protein. Owing to these limitations, the recombinant form of human Angpt1, though effective in mice, seems inapplicable for use in patients with sepsis.

In this proof-of-principle study, we could show for the first time that VT, a novel PEG-clustered synthetic 7-mer reported to activate Tie2 in endothelial cell culture and to promote angiogenesis in experimental diabetic ulcers, augments Tie2 activation *in vivo *and effectively protects against sepsis-induced vascular leakage and leukocyte transmigration in a clinically relevant murine model of polymicrobial abdominal sepsis. VT-driven effects were associated with significant improvements in several biochemical parameters of acute kidney and liver dysfunction. Most importantly, both pre- and post-treatment with VT significantly reduced mortality. The survival benefit seen with VT actually exceeded what we [6] and others [11] reported for recombinant Angpt1. A VT-like drug therefore may have great potential as a vascular barrier-protective agent in human sepsis as well as other conditions of acute vascular leakage and inflammatory endothelial dysfunction.

Increasing evidence suggests that Angpt1 attenuates endothelial barrier dysfunction and subsequent organ injury through multi-level effects on intracellular signaling, cytoskeleton, and junction-related molecules [10,23,35,36]. For example, operational Angpt1/Tie2 signaling prevents endothelial cell apoptosis by activating the phosphoinositol 3-kinase/Akt survival pathway [34,37]. Moreover, Angpt1 decreases vascular permeability through coordinated and opposite effects on the Rho GTPases Rac1 and RhoA, which in turn restrict the number and size of gaps that form at endothelial cell junctions in response to various leakage-inducing agents such as bradykinine, thrombin, VEGF, or TNF-α [10,23,35]. David and colleagues [38] recently showed that VT likewise counteracts endothelial gap formation and hyperpermeability induced by LPS or soluble mediators in pulmonary microvascular endothelial cells *in vitro*.

In addition, there is well-founded evidence that Angpt1 attenuates thrombin and VEGF-induced leukocyte adhesion and transmigration by blocking the upregulation of adhesion molecules ICAM-1, VCAM-1, and E-selectin on human umbilical vein endothelial cells [36,39]. Consistent with prior studies on Angpt1 overexpression in inflammatory models [6,7,9,11,40,41], the preventative effect of VT on leukocyte extravasation was associated with reduced expression of ICAM-1 and VCAM-1 in the present study. Although some investigators have described a role for Angpt1 in regulating chemotaxis and migration of human leukocytes [42-44], results from our complementary *in vitro *experiments suggest that VT is not sufficient to attenuate migratory capacity of murine PMNs.

Furthermore, as in some of the aforementioned studies, VT treatment prevented the upregulation of inflammatory cytokines and chemokines in serum and PL fluid. PMs not only are the most prominent leukocytes within the healthy peritoneal cavity but also play a central role in recruitment of blood-derived neutrophils in experimental peritonitis. Given that Angpt1 was recently found to block LPS-induced TNF-α production in macrophages specifically through Tie2 ligation [31], we hypothesized that the expression of ICAM-1 and VCAM-1 and subsequent PMN infiltration are regulated by macrophage-derived mediators in a Tie2-dependent fashion. Surprisingly, the release of various cytokines from LPS-treated macrophages *in vitro *was virtually not affected by VT whereas *in vivo *VT treatment led to a significant reduction in serum and PL fluid levels. We conclude that either VT can affect the proinflammatory mediator release from macrophages *in vivo *indirectly via activation of other cell types, which in turn can modulate PM responses, or these other cell types themselves may be responsible for the production of proinflammatory mediators during peritonitis. In the latter scenario, peritoneal mesothelial cells seem to be the most likely candidates as they have been shown to play a crucial role in initiating and amplifying the early inflammatory response to intra-abdominal infections [45]. However, Tie2 expression by mesothelial cells has not been reported yet. In summary, our findings suggest that VT exerts a preferential effect on the vascular endothelium-to which expression of Tie2 is largely limited.

Vascular barrier dysfunction has been shown to play a critical role in the complex pathogenesis of AKI [46]. VT-treated mice were protected from septic AKI as evidenced by preserved renal function parameters. However, our renal compartment-specific findings regarding adhesion molecule expression might mechanistically be involved but not exclusively responsible for the better organ function. In fact, the only modest suppression of ICAM-1 and VCAM-1 suggests that an improvement of other factors such as systemic and intrarenal hemodynamics might be accountable for the better renal outcome. Kim and colleagues [9] reported recently that pretreatment with an engineered variant of AdAng1 prevented the decrease in renal blood flow while improving the glomerular filtration rate in LPS-challenged mice.

Our preliminary studies to determine optimal VT dosing for the CLP studies revealed a biphasic response as determined by survival experiments. While these results were somewhat surprising, it was previously shown that high doses of VT result in a suboptimal activation of Tie2 receptor activity *in vitro *and decreased biological efficacy *in vivo *[26]. Experiments conducted to address potential reasons for this observation indicate that high concentrations of VT likely result in inefficient clustering of the Tie2 receptor monomers into high-order complexes [26]. Consistent with this notion, several published accounts demonstrate improved Tie2 activation to relatively low concentrations of Angpt1 versus higher concentrations in the same experimental setting [42,43,47,48]. Our findings emphasize, in the case of ligands directed at Tie2, the critical importance of not grossly oversaturating the system and thus impacting the ligand-driven clustering of Tie2. Furthermore, while every effort was made to deliver the most effective dose of VT, the dosing interval was based mainly on theoretical considerations. Preliminary experiments in healthy mice have shown that VT, in its PEGylated formulation, can sustain endothelial Tie2 activation for up to 72 hours (data not shown). Optimizing dosing intervals and treatment duration therefore could further improve outcome in experimental sepsis.

Solvent controls (in this case, PBS) are widely accepted in preclinical sepsis studies. While the most rigorous control for VT would have been a four-armed branched PEG attached to a scrambled peptide, we have to point out that these and similar controls have been examined previously [25,26]. Moreover, a 10-kDa, four-armed PEG (VT backbone) plus a cysteine on each arm did not activate Tie2 or improve outcome in murine endotoxemia [38].

Systemic exposure to LPS has been shown to suppress Tie2 protein abundance, Tie2 activation, and downstream signaling in murine lungs [6,49,50]. David and colleagues [38] recently demonstrated that the decreased lung Tie2 protein abundance during murine endotoxemia is overcome by increased activation of the remaining Tie2 molecules when VT is given. In the present study, we could essentially reproduce these findings in kidneys from septic CLP mice, suggesting that the loss of operational Tie2 signaling is a systemic event that occurs across various sepsis etiologies. It is noteworthy that VT was capable of augmenting Tie2 signaling through the remaining pool of receptors, thereby preserving net flux through this signaling pathway. However, it is possible that soluble Tie2, whose shedding probably depends on sepsis severity, scavenges VT and thereby ameliorates its effects on the vascular endothelium [51]. Thus, a likely therapeutic limitation is that VT has to be administered before the vascular damage is too grave to repair. In this regard, we were surprised that delayed administration of VT, as opposed to delayed transfection with AdAngpt1 [5], was still efficient at reducing mortality in the present study. Given these findings, it seems of utmost importance that VT be given as early as possible in order to maximize protective signaling through the remaining pool of Tie2 receptor molecules.

## Conclusions

In summary, we provide proof of principle in support of the efficacious use of PEGylated VT, a drug-like Tie2 receptor agonist, to activate Tie2 *in vivo*, counteract microvascular endothelial barrier dysfunction, improve multiple organ functions, and reduce mortality in a clinically relevant murine sepsis model. Further studies are needed to pave the road for clinical applications of this therapy concept.

## Key messages

• Systemic administration of vasculotide (VT) induced long-lasting Tie2 activation *in vivo*.

• VT protected against sepsis-induced vascular leakage and leukocyte transmigration.

• VT-driven effects were associated with significantly improved organ function and reduced circulating cytokine levels.

• Prophylactic as well as therapeutic VT treatment reduced mortality in septic mice.

## Abbreviations

AdAngpt1: agonist ligand angiopoietin-1; AKI: acute kidney injury; ALI: acute lung injury; Angpt1: angiopoietin-1; Angpt2: angiopoietin-2; BSA: bovine serum albumin; CLP: cecal ligation and puncture; ELISA: enzyme-linked immunosorbent assay; FCS: fetal calf serum; ICAM-1: intercellular adhesion molecule-1; IL-6: interleukin-6; i.p.: intraperitoneally; KC: keratinocyte chemoattractant; LPS: lipopolysaccharide; MCP-1: macrophage chemoattractant protein-1; MIP-2: macrophage inflammatory protein-2; PBS: phosphate-buffered saline; PEG: polyethylene glycol; PL: peritoneal lavage; PM: peritoneal macrophage; PMN: polymorphonuclear neutrophil; TNF-α: tumor necrosis factor-alpha; VCAM-1: vascular cell adhesion molecule-1; VEGF: vascular endothelial growth factor; VT: vasculotide.

## Competing interests

DJD and PVS are listed as inventors on two patents that are related to VT and that were filed by the Sunnybrook Research Institute. SMP is listed as an inventor on disclosures that regard angiopoietins and that were filed by Beth Israel Deaconess Medical Center. The laboratory of DJD has received a sponsored research agreement from a third-party company to conduct studies pertaining to the use of VT for diabetic wound healing. This party currently holds an option to license VT but in no way funded any of the work detailed in this article. The other authors declare that they have no competing interests.

## Authors' contributions

PK had the initial idea, supervised the project, analyzed the data, prepared the figures, and wrote the manuscript. FG, HH, and HP contributed to the design of the study and reviewed the manuscript. SD and SMP participated in interpreting the results and reviewed the manuscript. PVS and DJD generated the PEGylated Tie2 mimetic VT, performed *in vivo *experiments, and helped to write the manuscript. J-KP and CLB performed the histology and the immunofluorescence studies and helped to write the manuscript. NS designed and performed *in vivo *and *in vitro *experiments, analyzed the data, supervised the project, and wrote the manuscript. All authors read and approved the final manuscript.

## Supplementary Material

Additional file 1 **Vasculotide induces Tie2 posphorylation in the kidney in vivo**. A) Schematic of Vasculotide (VT): Four, eight amino acid peptides (NH2-CHHHRHSF-COOH) are covalently attached via cysteine and maleimide (denoted by X) to a 10 kDa, tetrameric polyethylene oxide. VT uses a peg to provide better stability, half-life, and resistance to immune reaction, but additionally, the 4-armed peg serves as a structural scaffold that displays 4 binding peptides in a configuration that is ideal for activating Tie2. The peptide (named T7) originally described by Tournaire R, et al (Ref 25) was found to bind outside of the shared Angpt1, Angpt2 ligand binding pocket and was thus not able to displace the endogenous ligands. This peptide was selected due to its reported high affinity and the desire to not displace endogenous ligands. In the initial publication describing VT (Ref 26) it was shown that unlike native Angpt1, VT is unable to bind integrins and activate associated downstream pathways. In its PEGylated form, the circulating half-life of VT appears to be around 24 hrs. B) Healthy mice (n = 3 per group) were euthanized at 16 h and 34 h after Vasculotide pre-treatment (200 ng Vasculotide i.p. at 0 h and 16 h). Mice (n = 3) injected with control buffer (PBS) only served as baseline controls (0 h). Immunoprecipitation (IP) for Tie2 and consecutive immunoblot (Blot) for phosphotyrosine (4G10) from kidney homogenates. C) Densitometry of B) showing the intensities of Tie2 tyrosine phosphorylation (pTyr/Tie2 ratio). Data are expressed as means ± SEM (*P < 0.05). Click here for file

Additional file 2 **Effect of Vasculotide on liver dysfunction**. Mice were pre-treated with Vasculotide (VT, 200 ng i.p.) or PBS at -16 h, -1 h prior to CLP or sham surgery. Blood samples were obtained at 18 hours (*n *= 10 per group) after CLP or sham treatment, respectively. Bar charts showing activity of A) aspartate aminotransferase (AST), and B) alanine aminotransferase (ALT). Data are expressed as means ± SEM (*n *= 7-10 mice/group). *P < 0.05; **P < 0.01 versus sham. †*P *< 0.05; ‡*P *< 0.01 versus CLP w/o VT. Click here for file

Additional file 3 **Vasculotide does not affect migratory capacity of PMN *in vitro***. A) Chemotaxis assessed by Transwell migration assays of bone marrow-derived neutrophils elicited with 300 ml of PL pools obtained at 18 h after surgery from seven to ten mice pre-treated with Vasculotide (VT, 200 ng i.p.) or PBS at -16 h, -1 h prior to CLP or sham surgery, respectively. B) Bone marrow-derived neutrophils were pre-incubated with different concentration of VT for 1 h and thereafter used for Transwell migration assays toward recombinant human C5a (50 ng/ml). C) Bone marrow-derived neutrophils were pre-incubated with different concentration of VT for 1 h and thereafter used for Transwell migration assays toward 300 ml of PL pools obtained at 18 h after CLP-surgery. Data are presented as the percentage of PMN number loaded into the upper chamber that had migrated to the bottom well, expressed as means ± SEM (two independent experiments performed in duplicates). *P < 0.05; **P < 0.01 vs. sham. †*P *< 0.05; ‡*P *< 0.01 vs. CLP w/o VT. Click here for file
