# Supplementary figures and images for: The synthetic Tie2 agonist peptide vasculotide protects against vascular leakage and reduces mortality in murine abdominal sepsis
Source: Crit Care. 2011 Oct 31;15(5):R261. doi: 10.1186/cc10523 (PMC3334812; doi:10.1186/cc10523)

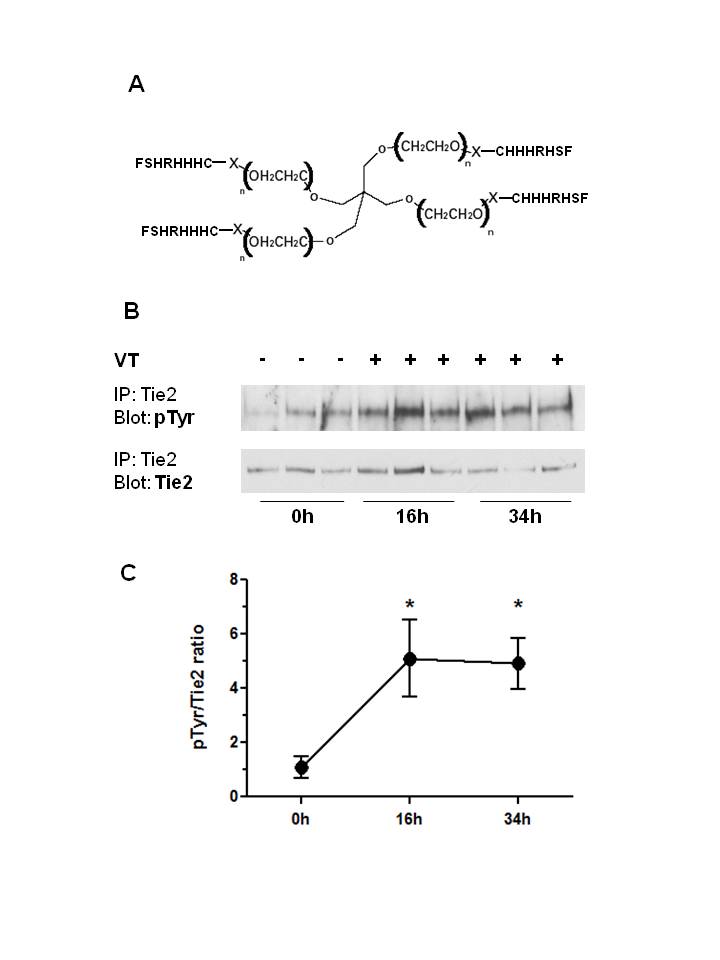

Supplement: Additional file 1 — Vasculotide induces Tie2 posphorylation in the kidney in vivo. A) Schematic of Vasculotide (VT): Four, eight amino acid peptides (NH2-CHHHRHSF-COOH) are covalently attached via cysteine and maleimide (denoted by X) to a 10 kDa, tetrameric polyethylene oxide. VT uses a peg to provide better stability, half-life, and resistance to immune reaction, but additionally, the 4-armed peg serves as a structural scaffold that displays 4 binding peptides in a configuration that is ideal for activating Tie2. The peptide (named T7) originally described by Tournaire R, et al (Ref 25) was found to bind outside of the shared Angpt1, Angpt2 ligand binding pocket and was thus not able to displace the endogenous ligands. This peptide was selected due to its reported high affinity and the desire to not displace endogenous ligands. In the initial publication describing VT (Ref 26) it was shown that unlike native Angpt1, VT is unable to bind integrins and activate associated downstream pathways. In its PEGylated form, the circulating half-life of VT appears to be around 24 hrs. B) Healthy mice (n = 3 per group) were euthanized at 16 h and 34 h after Vasculotide pre-treatment (200 ng Vasculotide i.p. at 0 h and 16 h). Mice (n = 3) injected with control buffer (PBS) only served as baseline controls (0 h). Immunoprecipitation (IP) for Tie2 and consecutive immunoblot (Blot) for phosphotyrosine (4G10) from kidney homogenates. C) Densitometry of B) showing the intensities of Tie2 tyrosine phosphorylation (pTyr/Tie2 ratio). Data are expressed as means ± SEM (*P < 0.05). [file cc10523-S1.JPEG]

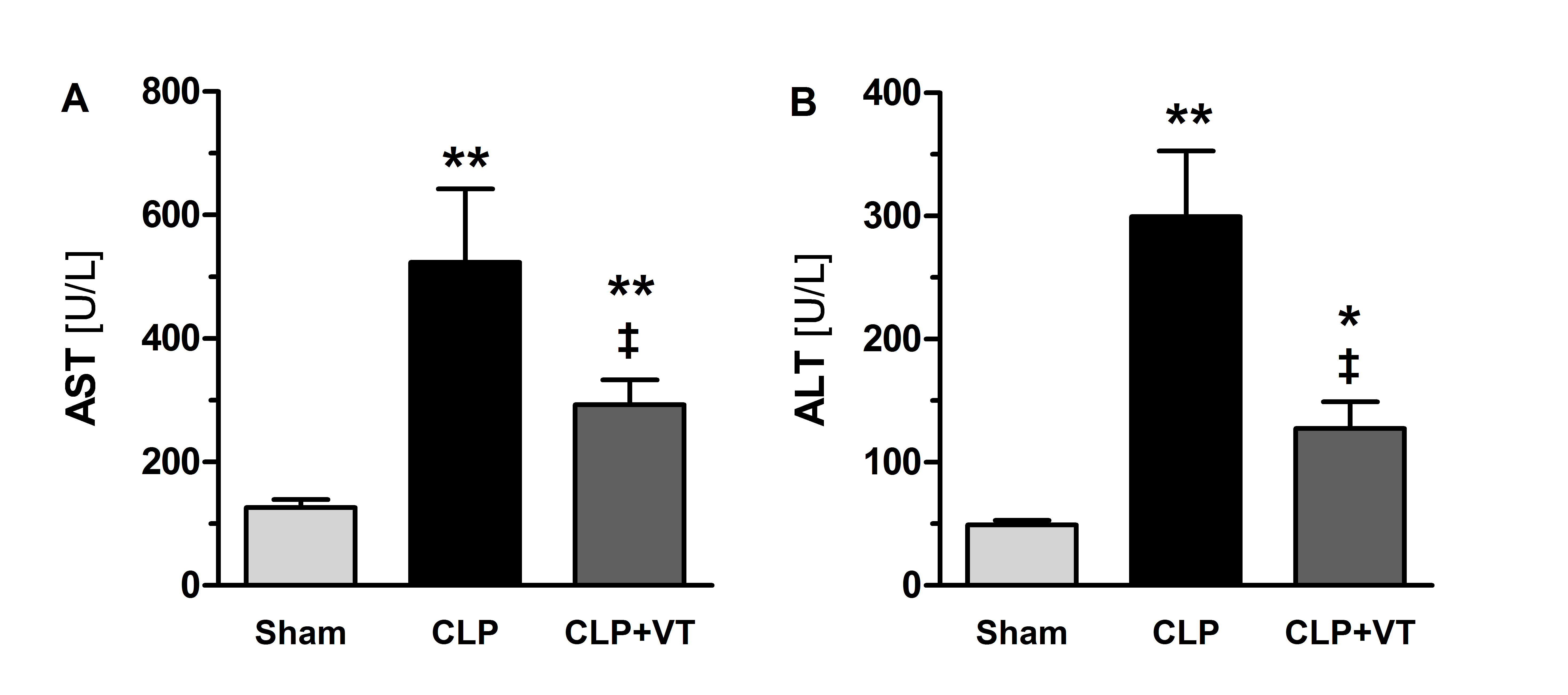

Supplement: Additional file 2 — Effect of Vasculotide on liver dysfunction. Mice were pre-treated with Vasculotide (VT, 200 ng i.p.) or PBS at -16 h, -1 h prior to CLP or sham surgery. Blood samples were obtained at 18 hours (n = 10 per group) after CLP or sham treatment, respectively. Bar charts showing activity of A) aspartate aminotransferase (AST), and B) alanine aminotransferase (ALT). Data are expressed as means ± SEM (n = 7-10 mice/group). *P < 0.05; **P < 0.01 versus sham. †P < 0.05; ‡P < 0.01 versus CLP w/o VT. [file cc10523-S2.JPEG]

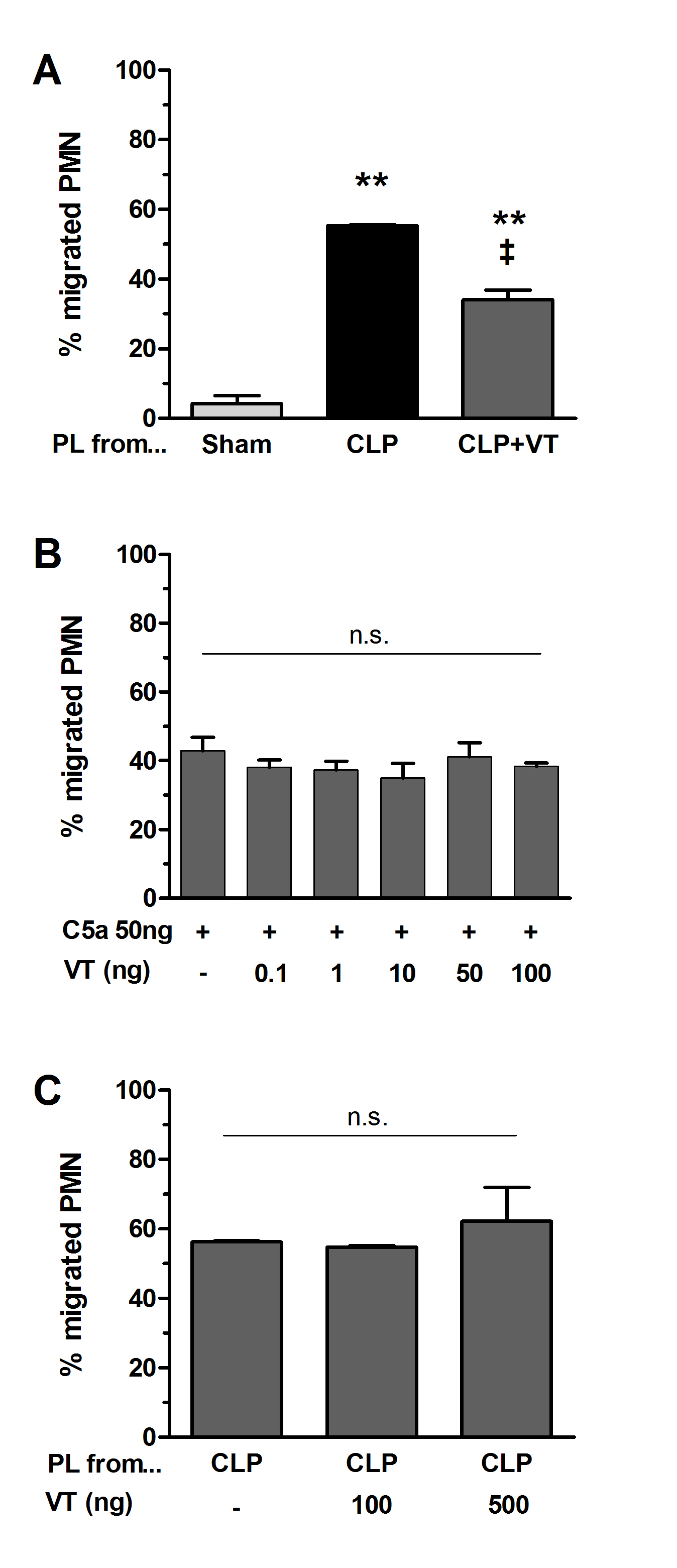

Supplement: Additional file 3 — Vasculotide does not affect migratory capacity of PMN in vitro. A) Chemotaxis assessed by Transwell migration assays of bone marrow-derived neutrophils elicited with 300 ml of PL pools obtained at 18 h after surgery from seven to ten mice pre-treated with Vasculotide (VT, 200 ng i.p.) or PBS at -16 h, -1 h prior to CLP or sham surgery, respectively. B) Bone marrow-derived neutrophils were pre-incubated with different concentration of VT for 1 h and thereafter used for Transwell migration assays toward recombinant human C5a (50 ng/ml). C) Bone marrow-derived neutrophils were pre-incubated with different concentration of VT for 1 h and thereafter used for Transwell migration assays toward 300 ml of PL pools obtained at 18 h after CLP-surgery. Data are presented as the percentage of PMN number loaded into the upper chamber that had migrated to the bottom well, expressed as means ± SEM (two independent experiments performed in duplicates). *P < 0.05; **P < 0.01 vs. sham. †P < 0.05; ‡P < 0.01 vs. CLP w/o VT. [file cc10523-S3.JPEG]
